# Therapeutic Balloon‐Assisted Enteroscopy: Recent Advances in the Management of Small Bowel Diseases

**DOI:** 10.1002/deo2.70449

**Published:** 2026-10-08

**Authors:** Manabu Nagayama, Tsubasa Kinoshita, Hiroyuki Kawaguchi, Toshihiro Fujinuma, Sawako Fujikura, Hiroki Hayashi, Tomoko Tamaru, Hirotsugu Sakamoto, Tomonori Yano

**Affiliations:** ^1^ Department of Medicine Division of Gastroenterology, Jichi Medical University Tochigi Japan; ^2^ Department of Endoscopic Research and International Education Funded by FUJIFILM Medical Co. Ltd. Jichi Medical University Shimotsuke Japan

**Keywords:** Crohn's disease, double‐balloon enteroscopy, familial adenomatous polyposis, gastrointestinal hemorrhage, Peutz‐Jeghers syndrome

## Abstract

Balloon‐assisted enteroscopy (BAE) has substantially advanced small bowel disease management. This review summarizes recent therapeutic BAE advances, focusing on three major indications: small bowel vascular lesions, hereditary polyposis syndromes, and Crohn's disease (CD). For suspected small bowel bleeding from vascular lesions, morphological classification guides lesion‐specific hemostasis—primarily using argon plasma coagulation and clipping—while gel immersion endoscopy helps secure the visual field. In hereditary polyposis syndromes (e.g., Peutz–Jeghers syndrome and familial adenomatous polyposis), endoscopic ischemic and cold snare polypectomy prevent intussusception and reduce cancer risk, decreasing surgical needs. Additionally, endoscopic balloon dilation is an established bowel‐preserving treatment for fibrotic CD strictures, reducing surgical resection and short bowel syndrome risk. Driven by evolving imaging and devices, BAE remains central to small bowel management, improving patient outcomes and quality of life.

## 1 Introduction

Diagnostic modalities for evaluating the small bowel have become increasingly diverse. These include imaging modalities such as computed tomography (CT), magnetic resonance imaging (MRI), and abdominal ultrasonography, as well as endoscopic modalities such as balloon‐assisted enteroscopy (BAE) and small bowel capsule endoscopy (SBCE). Consequently, a wealth of knowledge regarding small bowel diseases has been acquired, leading to a paradigm shift in their diagnostic and therapeutic strategies.

Endoscopic therapeutic procedures using BAE are wide‐ranging, including endoscopic submucosal dissection and endoscopic injection sclerotherapy [1, 2, 3]. This article reviews the outlines of clinical management strategies and the role of small bowel BAE therein, focusing primarily on small‐bowel vascular lesions (SBVLs), Peutz‐Jeghers syndrome (PJS) and familial adenomatous polyposis (FAP), and small bowel strictures associated with Crohn's disease (CD).

## 2 Devices and Methods for Therapeutic Enteroscopy

Therapeutic BAE features a working length of 2,000 mm, an outer diameter at the distal end of 9.2–9.4 mm, and a working channel diameter of 3.2 mm, which facilitates the use of various therapeutic accessories. BAE includes double‐balloon enteroscopy (DBE) and single‐balloon enteroscopy (SBE). DBE uses balloons on both the endoscope and overtube, which may provide superior anchoring and more stable insertion, whereas SBE uses a single overtube balloon, allowing relatively simple setup [4]. The latest therapeutic DBE, EN‐840T (FUJIFILM, Tokyo, Japan), has a 9.4‐mm distal‐end diameter and features a high‐resolution CMOS sensor, dedicated water jet, linked color imaging (LCI), and blue light imaging (BLI). The current SBE, SIF‐H190 (Olympus, Tokyo, Japan), has a 9.2‐mm distal‐end diameter and supports narrow band imaging (NBI), texture and color enhancement imaging (TXI), and red dichromatic imaging (RDI) with EVIS X1. Short‐length endoscopes are also available: the EI‐580BT (FUJIFILM; working length, 1550 mm) and SIF‐H290S (Olympus; 1520 mm).

Sedation depth depends on the insertion route. At our institution, transanal enteroscopy is generally performed under moderate (conscious) sedation, whereas transoral enteroscopy requires deeper sedation because of pharyngeal discomfort. Pethidine hydrochloride and midazolam are commonly used, with flunitrazepam, remimazolam, or propofol selected when appropriate. Propofol may be selected when a more predictable sedation depth or rapid recovery is desired. Timepidium bromide hydrate or glucagon is used as an antispasmodic according to comorbidities. Vital signs and respiratory status are monitored during sedation, and patients are observed until adequate recovery. Outpatients receiving sedatives are instructed not to drive for the remainder of the day.

## 3 Hemostasis for Vascular Lesions

### 3.1 Definition of Terms for Small Bowel Bleeding and Diagnostic/Therapeutic Algorithms

The 2015 American guideline redefined obscure gastrointestinal bleeding (OGIB) as a condition where the bleeding source cannot be identified anywhere in the gastrointestinal tract, including the small bowel. Instead, the term small bowel bleeding (SBB) has been proposed to replace the previous term OGIB, which refers to bleeding that persists or recurs after a negative bidirectional endoscopic evaluation, including upper endoscopy and colonoscopy [5]. Following this approach, the 2023 European guideline utilized the term suspected SBB (SSBB) [6]. The second edition of the Japanese Guideline for Small Bowel Endoscopy, to be published in 2026, also adopts a similar definition [7].

Regarding investigations for SSBB, Western guidelines recommend SBCE as the first‐line modality for small bowel evaluation [5, 6, 8]. Imaging modalities such as CT are positioned as complementary examinations when SBCE findings are negative, when a tumor is suspected, or when SBCE is contraindicated (e.g., suspected strictures). Conversely, the Japanese guidelines recommend performing a CT as the first‐line modality [7]. This is partly attributable to the high availability of CT scanners, which facilitates rapid evaluation in both elective and emergency settings [9]. Dynamic CT imaging is considered essential for diagnosing subepithelial tumors that are difficult to detect via SBCE (such as gastrointestinal stromal tumors), as well as arteriovenous malformations (AVMs) and varices. When abnormal findings such as active contrast extravasation or neoplastic lesions are detected, BAE is indicated, and these findings help guide the selection of the insertion route.

### 3.2 Vascular Lesions and Risk Assessment

Major risk factors for SBVLs include aging, chronic renal failure, liver cirrhosis, and cardiac diseases such as valvular heart disease [5, 7, 10]. Notably, the clinical condition in which aortic stenosis is complicated by gastrointestinal bleeding is known as Heyde syndrome [5]. Furthermore, genetic backgrounds such as hereditary hemorrhagic telangiectasia are rarely involved [5, 7, 11]. The Ohmiya index is a comorbidity‐based scoring system that helps predict whether the source of SBB is of vascular or nonvascular origin [7, 12].

### 3.3 Management of Vascular Lesions via BAE

There are various scenarios in which BAE is indicated for SSBB. For instance, when a lesion responsible for the bleeding (such as a vascular lesion, tumor, or ulcer) is identified by SBCE or CT, BAE is performed with the intent of endoscopic treatment. Furthermore, even in the absence of CT abnormalities, BAE is undertaken if SBVLs are strongly suspected. Additionally, BAE is considered when bleeding persists or recurs despite negative SBCE findings, provided that a small bowel lesion is clinically highly suspected. It is also indicated in cases of overt bleeding (such as hematochezia or melena) where immediate simultaneous diagnosis and hemostatic treatment are required.

For SBB, a cylindrical transparent hood is considered the most suitable distal attachment. The reasons include improved identification rates of SBVLs, securement and stabilization of the visual field, and enhanced scope insertability [13, 14]. Moreover, even within a narrow lumen, the cylindrical transparent hood secures adequate space, thereby facilitating the application of hemostatic clips.

Regarding the selection of the insertion route when performing BAE for SBB, the transoral route is generally prioritized in cases of overt bleeding [7]. This preference is based on the fact that transoral insertion achieves a significantly longer insertion length than transanal insertion. Furthermore, during massive hemorrhage, securing a clear visual field via the transanal route is difficult due to passage through an inadequately prepared colon; conversely, the transoral route can be performed with fasting alone, and estimating the source of bleeding is relatively easy as the luminal contents become increasingly bloody upon approaching the bleeding site. Additionally, the color of bloody stool serves as a clue for estimating the site of bleeding: the transoral route should be considered for melena, whereas the transanal route should be considered for hematochezia. However, caution is required because massive hemorrhage from the upper jejunum can also present as hematochezia. If prior investigations such as SBCE or imaging modalities have been performed, the insertion route should be selected based on those findings.

In SBB, it is crucial to perform scope insertion with as little insufflation as possible—using carbon dioxide—and to avoid washing away blood with excessive water jet or irrigation to reliably identify the lesion [7, 15]. During transoral insertion, when bloody intestinal fluid is encountered, the bleeding source is assumed to be nearby. In such cases, a clip should be placed as a marker to facilitate subsequent orientation. When securing a clear visual field is difficult due to massive hemorrhage, performing gel immersion endoscopy facilitates the identification of the bleeding point [15]. The procedure for gel immersion endoscopy involves sufficiently aspirating intraluminal gas prior to injection. Then, a transparent gel with appropriate viscosity, such as Viscoclear (Otsuka Pharmaceutical Factory, Tokushima, Japan), is injected through the working channel of the endoscope. Inflating the balloon at the endoscope tip or on the overtube prevents backflow of the gel, allowing for the efficient displacement of blood and debris.

Endoscopic hemostasis using BAE for SBB is an established therapeutic modality that provides high success rates (40%–73%) and clinical improvement [5, 16]. Because the small bowel is characterized by an extremely thin intestinal wall, it is recommended to select a technique tailored to the lesion findings while minimizing the risk of perforation. The two primary recommended hemostatic methods are argon plasma coagulation (APC) and clipping [7]. The characteristics of APC, which allows for “wide and shallow” tissue coagulation, are well‐suited to the anatomical features of the small bowel, making it the most widely used first‐line therapy globally for angioectasia. Clipping is also utilized as widely as APC, as it carries a lower risk of perforation because it does not involve thermal coagulation.

The Yano‐Yamamoto classification for SBVLs categorizes lesions based on morphology and pulsation during real‐time observation via DBE [17]. Utilizing this classification enables the differentiation between venous and arterial lesions, allowing for the selection of an appropriate hemostatic method [18]. For venous and capillary lesions (angioectasia, Types 1a and 1b), APC is the first choice (Figure 1a,b). For lesions of Type 1b, there is a risk of damaging the muscular layer due to prolonged coagulation time; therefore, a technique combining APC with the submucosal injection of saline or epinephrine solution has also been proposed [18]. For arterial lesions causing pulsatile bleeding (Dieulafoy's lesions [Types 2a and 2b] and AVMs [Type 3]), mechanical hemostasis using clipping is strongly recommended [17, 18]. In cases of giant AVMs or when endoscopic access and hemostasis are difficult, a prompt transition to surgical resection or interventional radiology via transcatheter arterial embolization should be made [7]. In such scenarios, endoscopic marking with tattooing or clips is useful to facilitate localization of the lesion. For angioectasia causing hemorrhage, excision of the angioectasia using a cold snare without coagulation, followed by clipping for hemostasis, has also been reported (Figure 1c–f) [19].

**FIGURE 1:**
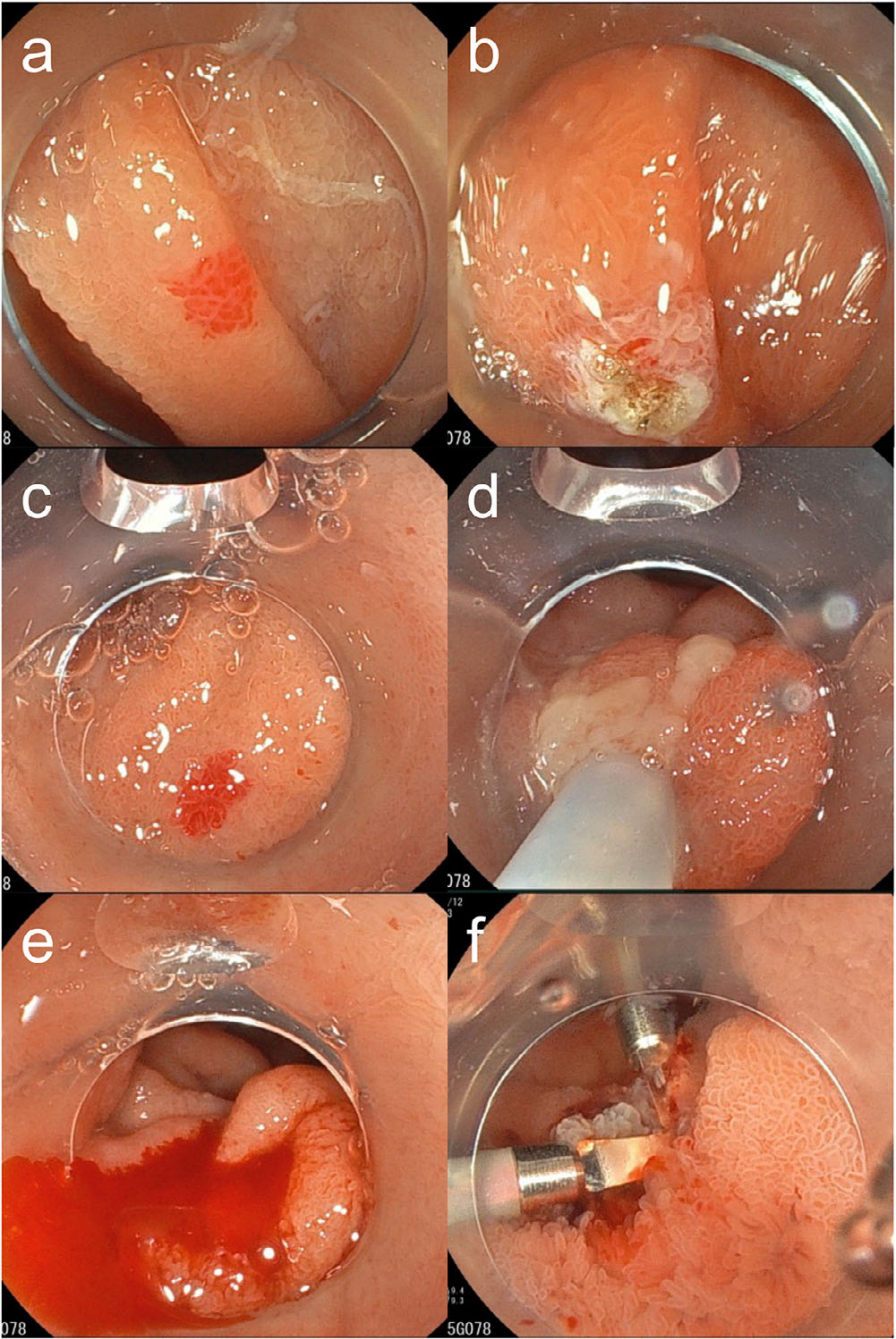
Endoscopic treatment of small bowel vascular lesions using balloon‐assisted enteroscopy (BAE). (a, b) A Type 1b angioectasia treated with argon plasma coagulation (APC). (c–f) A Type 1b angioectasia treated by the cold snare technique. (d) Resection of the lesion using the cold snare technique. (e) Immediate post‐resection bleeding. (f) Ligation of the feeding vessel with two endoscopic clips.

## 4 Endoscopic Treatment via BAE for Small Bowel Polyposis

### 4.1 PJS and FAP

The advent of enteroscopy, particularly BAE, has enabled minimally invasive endoscopic treatment for small bowel polyps in hereditary polyposis syndromes, which were previously managed by surgical resection.

PJS is a rare disease characterized by hamartomatous polyposis throughout the entire gastrointestinal tract except the esophagus, accompanied by mucocutaneous pigmentations on the lips, fingers, and other areas [7, 20]. It is an autosomal dominant hereditary disorder caused by pathogenic germline variants in STK11; however, approximately 17%–50% of cases are sporadic with no family history. Ninety‐six percent of the polyps develop in the small bowel, and enlarged polyps cause intussusception, gastrointestinal bleeding, and intestinal obstruction from a young age, often necessitating surgical resection. Furthermore, it carries a high lifelong risk of malignant tumors developing not only in the gastrointestinal tract but also in other organs.

FAP is an autosomal dominant disorder primarily caused by pathogenic germline variants in APC [21, 22, 23]. Hundreds to thousands of adenomas develop throughout the large intestine, progressing to colorectal cancer in nearly 100% of cases if left untreated. Adenomas also frequently develop in the extra‐colonic gastrointestinal tract, particularly in the duodenum and the upper small bowel. The risk of developing small bowel cancer is known to be significantly higher than that in the general population [21, 22].

In both diseases, polyps are known to develop throughout the entire small bowel, though they tend to occur more frequently in the jejunum [7, 20, 21, 22]. Initial evaluation requires accurate assessment of lesion distribution, number, and size using imaging modalities such as CT and endoscopic modalities such as SBCE and BAE to formulate appropriate therapeutic strategies. Regarding the distal attachment used in BAE, a cylindrical transparent hood is the most suitable, for the same reasons described in the section on SBB.

### 4.2 BAE Treatment for Small Bowel Polyps in PJS

The primary objective of small bowel management in PJS is the prevention of intussusception and the subsequent avoidance of surgical intervention [7, 20]. Once a surgical procedure is performed, postoperative adhesions develop, making subsequent deep insertion via BAE difficult; therefore, managing these lesions through endoscopic resection is considered desirable. The guidelines recommend that the initial gastrointestinal evaluation in patients with PJS should be performed by the age of 8 years even in the absence of symptoms, and if polyps are detected, surveillance should be conducted every 1–3 years [20, 21, 22]. The examination interval needs to be determined based on the growth rate of the polyps. Furthermore, because small bowel polyps exceeding 15 mm can trigger intussusception, treatment via BAE is recommended for polyps measuring 10 mm or larger [7]. Repetitive endoscopic polypectomy has been shown to reduce the number of polyps, highlighting the importance of continuous monitoring [24]. Proactive intervention with BAE has been proven to dramatically reduce the frequency of emergency surgeries and bowel resections in patients with PJS [24, 25, 26, 27].

Conventionally, the treatment of polyps in PJS has focused primarily on conventional polypectomy and endoscopic mucosal resection (EMR). To safely manage multiple polyps within a short period, endoscopic ischemic polypectomy (EIP) was developed in Japan. In this technique, the stalk of the polyp is ligated with a clip or a detachable snare to induce ischemic necrosis and subsequent sloughing of the lesion (Figure 2a–e) [28, 29, 30]. Compared with conventional polypectomy, EIP has a significantly lower complication rate and offers the advantage of allowing the treatment of a larger number of lesions during a single procedure [31, 32]. When the ischemic effect is successfully achieved, a change in color to purple is observed within 1–2 min. It has been reported that when ligation with a single clip fails to induce sufficient ischemia, the application of an additional clip from a different direction, a technique known as crossed‐clip strangulation, may achieve more effective ischemic necrosis [29]. However, because specimen retrieval is impossible with EIP, lesions suspicious for malignancy should be resected via conventional polypectomy, or a portion of the polyp should be transected after EIP for histological evaluation.

**FIGURE 2:**
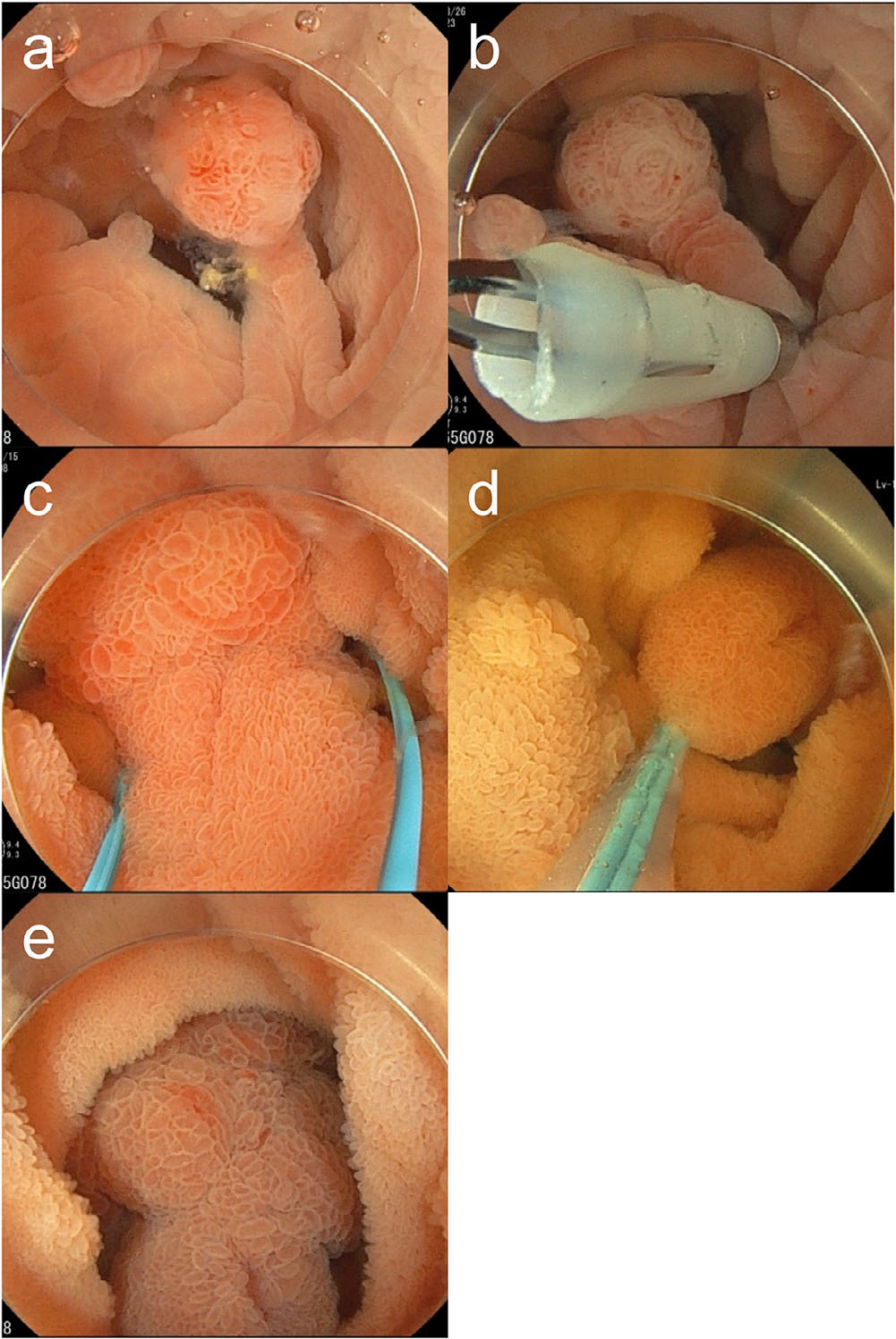
Endoscopic ischemic polypectomy (EIP) for small bowel polyps in Peutz–Jeghers syndrome (PJS) using balloon‐assisted enteroscopy (BAE). (a, b) EIP using clips for a 10‐mm Ip polyp. (c–e) EIP using a detachable snare for a 25‐mm Ip polyp. (c) Ischemic discoloration of the polyp following successful ligation.

### 4.3 BAE Treatment for Small Bowel Intussusception in PJS

PJS frequently causes intussusception with a small bowel polyp acting as the lead point; a multicenter survey in Japan reported that intussusception was observed in 67.7% of cases [27]. When a patient with PJS complains of bowel obstruction symptoms such as abdominal pain or nausea, evaluation should be performed using contrast‐enhanced CT. Upon confirming intussusception, its location, polyp size, and blood flow impairment must be evaluated. If findings requiring surgery are absent, endoscopic reduction of intussusception via DBE should be considered [33, 34]. During transoral DBE, the minimal water exchange method is used to minimize intraluminal pressure, and the intussusception is recognized as a narrowed lumen with deeply invaginated mucosa (Figure 3a–d) [31, 35, 36]. After traversing the intussusception, the endoscope is advanced approximately 10 cm beyond the lesion, the endoscope balloon is inflated, and gentle withdrawal is performed to achieve reduction [31, 33]. For transanal DBE, after reaching the leading polyp, the endoscope balloon is inflated, and water or contrast medium is injected to achieve reduction of the intussusception [34]. In either approach, after the intussusception is successfully reduced, the culprit polyp can be immediately removed during the same endoscopic session. The efficacy of this endoscopic reduction technique is favorable at 96% (24/25 lesions); adve

**FIGURE 3:**
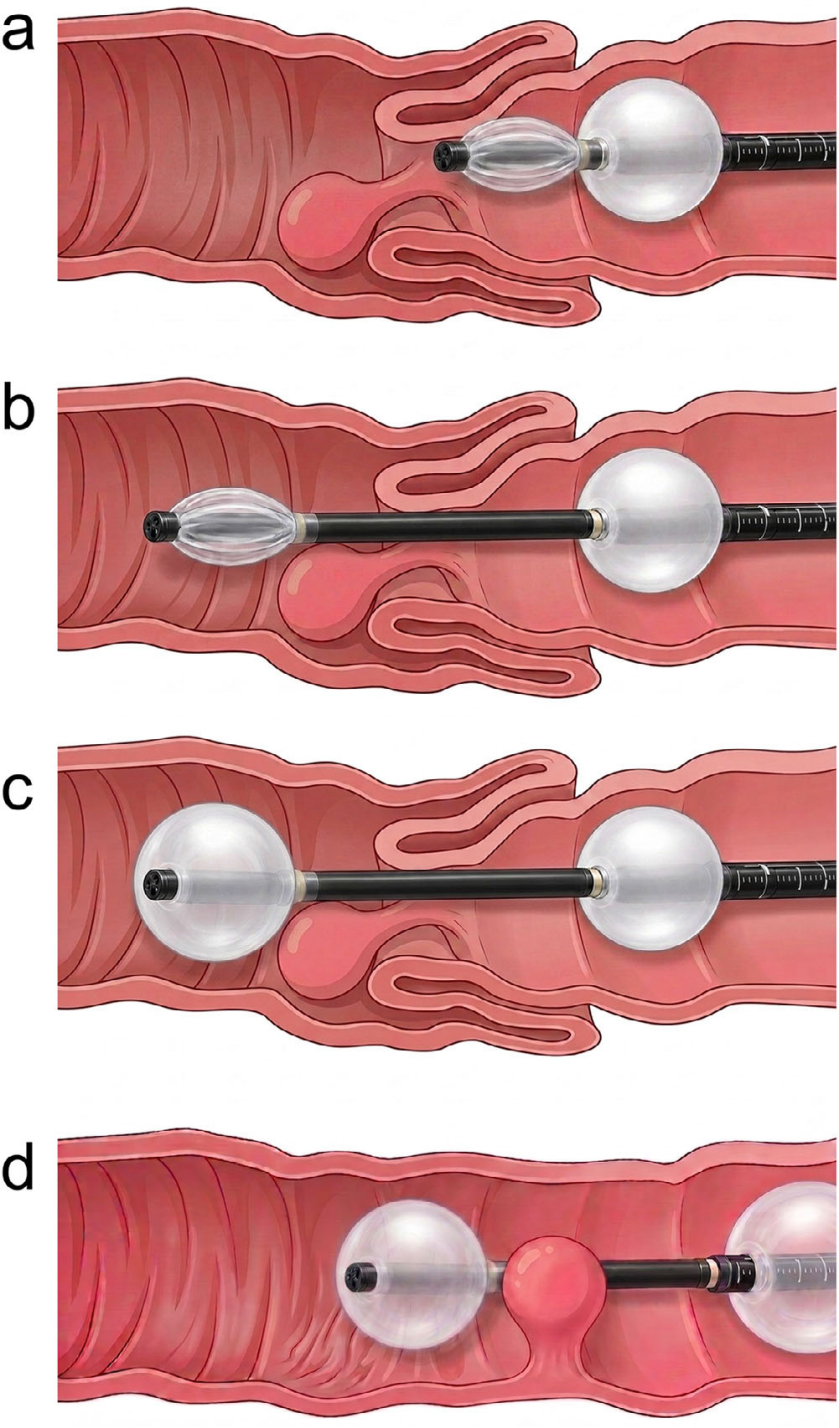
Endoscopic reduction of intussusception caused by a small bowel polyp in Peutz–Jeghers syndrome (PJS) using the transoral approach. (a) The intussusception is identified as a narrowed lumen with deeply invaginated small bowel mucosa. (b) After careful passage beyond the intussusception and leading polyp, the normal lumen becomes visible. (c) The endoscope is advanced approximately 10 cm beyond the leading polyp, and the distal balloon is moderately inflated. (d) Gentle withdrawal of the endoscope with the balloon inflated results in reduction of the intussusception.

### 4.4 BAE Treatment for Small Bowel Polyps in FAP

In the post‐colectomy management of patients with FAP, duodenal and deep small‐bowel adenomas represent a major clinical challenge. Although duodenal adenomas are assessed using the Spigelman classification, patients with Spigelman stage III or IV disease have a higher prevalence of jejunal and ileal adenomas [38, 39]. For duodenal adenomas associated with FAP, proactive resection using underwater EMR and cold snare polypectomy (CSP) is performed as intensive downstaging polypectomy to prevent malignant transformation (Figure 4a–d) [40, 41]. Similar management strategies are being applied to deep small‐bowel adenomas. In a series of FAP‐associated jejunal adenomas ≥5 mm treated by DBE‐assisted CSP, all resected lesions were histologically confirmed as adenomas, with no post‐procedural bleeding reported [42]. This indicates that CSP may be a safe and useful technique for deep small bowel adenomas associated with FAP.

**FIGURE 4:**
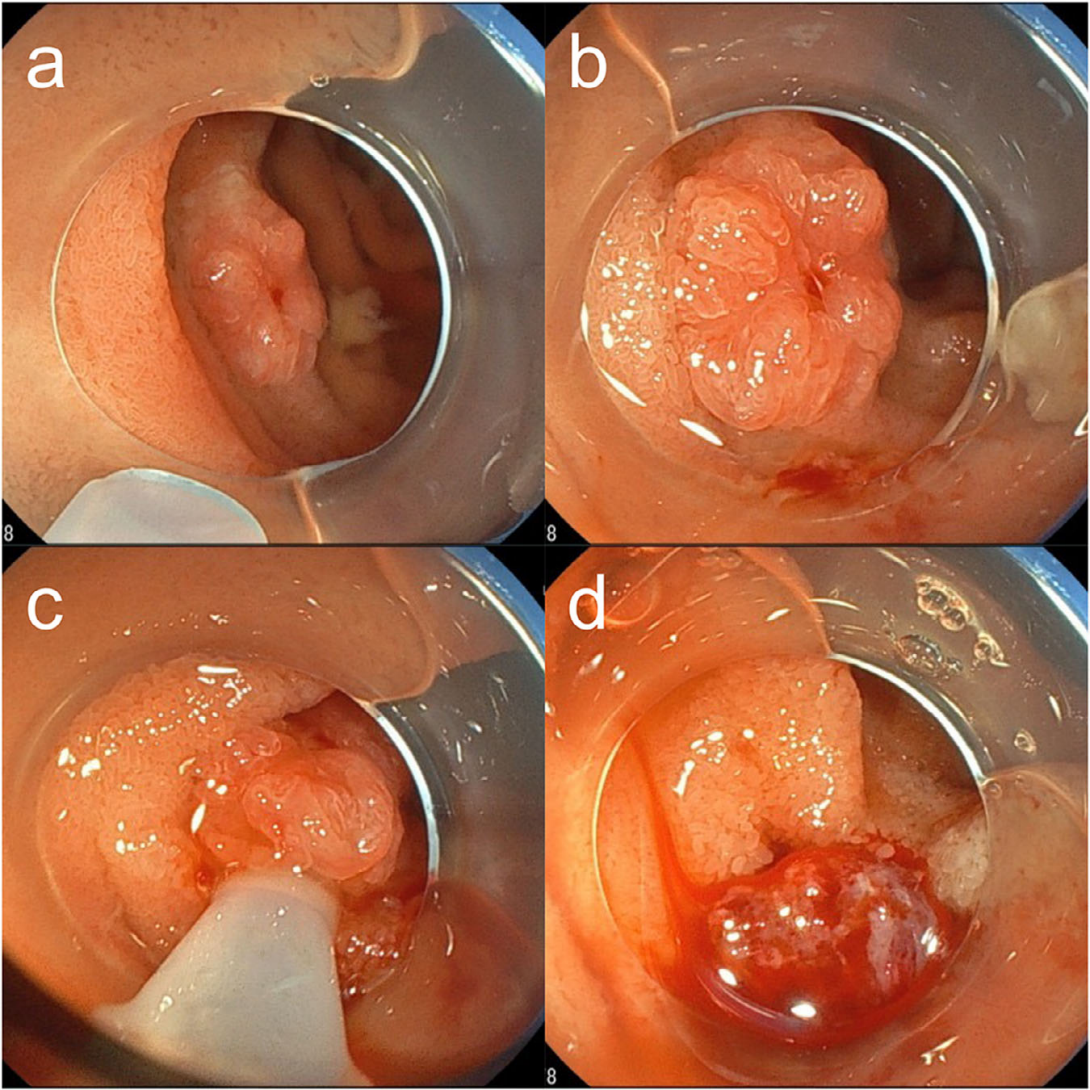
Cold snare polypectomy (CSP) for an 8‐mm type IIa adenoma in familial adenomatous polyposis (FAP). (a, b) An 8‐mm type IIa adenoma in the jejunum. (c, d) Resection of the lesion using CSP.

Although evidence remains limited regarding the impact of endoscopic treatment of FAP‐associated small bowel lesions on cancer prevention and prognosis, periodic whole‐small‐bowel surveillance with BAE and appropriate endoscopic resection may become the standard of care for patients with advanced duodenal disease [7, 21, 43, 44].

## 5 Balloon Dilation for Small Bowel Strictures in CD

### 5.1 Overview of Small Bowel Strictures in CD

CD is characterized by chronic transmural inflammation of the gastrointestinal tract wall, frequently leading to complications such as intestinal strictures and fistulas [45, 46]. Small bowel strictures not only cause obstructive symptoms and impaired quality of life but also constitute a major indication for surgery. Population‐based studies have shown that 5%–28% of patients with CD have stricturing disease at diagnosis, and more than 50% develop stricturing complications within 10 years [47].

Small bowel strictures are broadly classified as inflammatory or fibrotic. The former results from active inflammation and edema, whereas the latter is characterized by collagen deposition and irreversible bowel wall fibrosis [47, 48]. In clinical practice, inflammatory and fibrotic components frequently coexist, making precise distinction challenging. Nevertheless, endoscopic balloon dilation (EBD) is generally indicated for predominantly fibrotic strictures after inflammation has been adequately controlled with medical therapy [6, 47, 49]. As another therapeutic option, endoscopic radial incision and cutting have been reported [50]. Leaving strictures untreated leads to intestinal obstruction and malnutrition, increasing the risk of emergency surgery; thus, intervention at an appropriate time is crucial.

### 5.2 Indications and Exclusion Criteria for EBD

Several reports describe the indication criteria, including symptomatic fibrotic strictures after medical therapy or asymptomatic strictures accompanied by proximal bowel dilation [51, 52]. Generally, short strictures with a longitudinal length of 5 cm or less are considered the ideal indication [53, 54]. It has been reported that each 1‐cm increase in stricture length raises the future surgical rate by 8%, indicating that shorter lesions tend to benefit more from EBD [55].

Conversely, exclusion criteria have been established because of the increased risk of serious complications, including active inflammation accompanied by deep ulcerations extending to the muscular layer [49]. Furthermore, the European Crohn's and Colitis Organization consensus considers strictures accompanied by fistulas or abscesses, as well as malignant strictures containing high‐grade dysplasia, as contraindications for EBD [53]. In Japan, the presence of fistulas or abscesses is considered an exclusion criterion for EBD in some studies, whereas others have included patients with internal fistulas. Nevertheless, internal fistulas have been identified as a risk factor for subsequent surgery [49, 52]. Additionally, EBD should be avoided in the presence of marked angulation or adhesions because these conditions increase the risk of perforation during balloon dilation. Severe malnutrition and poor performance status should also be considered when determining the appropriateness of EBD versus surgical treatment [56].

### 5.3 Technique of EBD via BAE

Once clinical remission has been achieved, BAE‐guided EBD is planned. Because CD predominantly affects the ileum, the transanal approach is generally preferred; however, the transoral approach may be advantageous in patients with adhesions or bowel deformity. For transanal BAE, standard colonoscopy bowel preparation is performed, with the cleansing solution administered slowly in divided doses to reduce the risk of obstruction. The CAST hood (TOP Co., Tokyo, Japan) allows accurate measurement of stricture diameter and facilitates appropriate balloon selection (Figure 5a). Furthermore, it offers advantages such as facilitating guidewire insertion into severe strictures and easing the passage of the scope through the stricture after dilation [7, 13]. The dilation balloons used are generally through‐the‐scope balloons. Additionally, it is safer to use a guidewire when advancing the dilation balloon into the deep intestine under fluoroscopic guidance. The minimal water exchange method is useful during BAE for CD [35]. This technique uses minimal water infusion to maintain a clear visual field, allowing endoscope advancement while minimizing bowel distension and intraluminal pressure [57]. In addition to facilitating bowel shortening and reducing post‐procedural discomfort, this method enables excellent contrast‐filling images during endoscopic selective enteroclysis.

**FIGURE 5:**
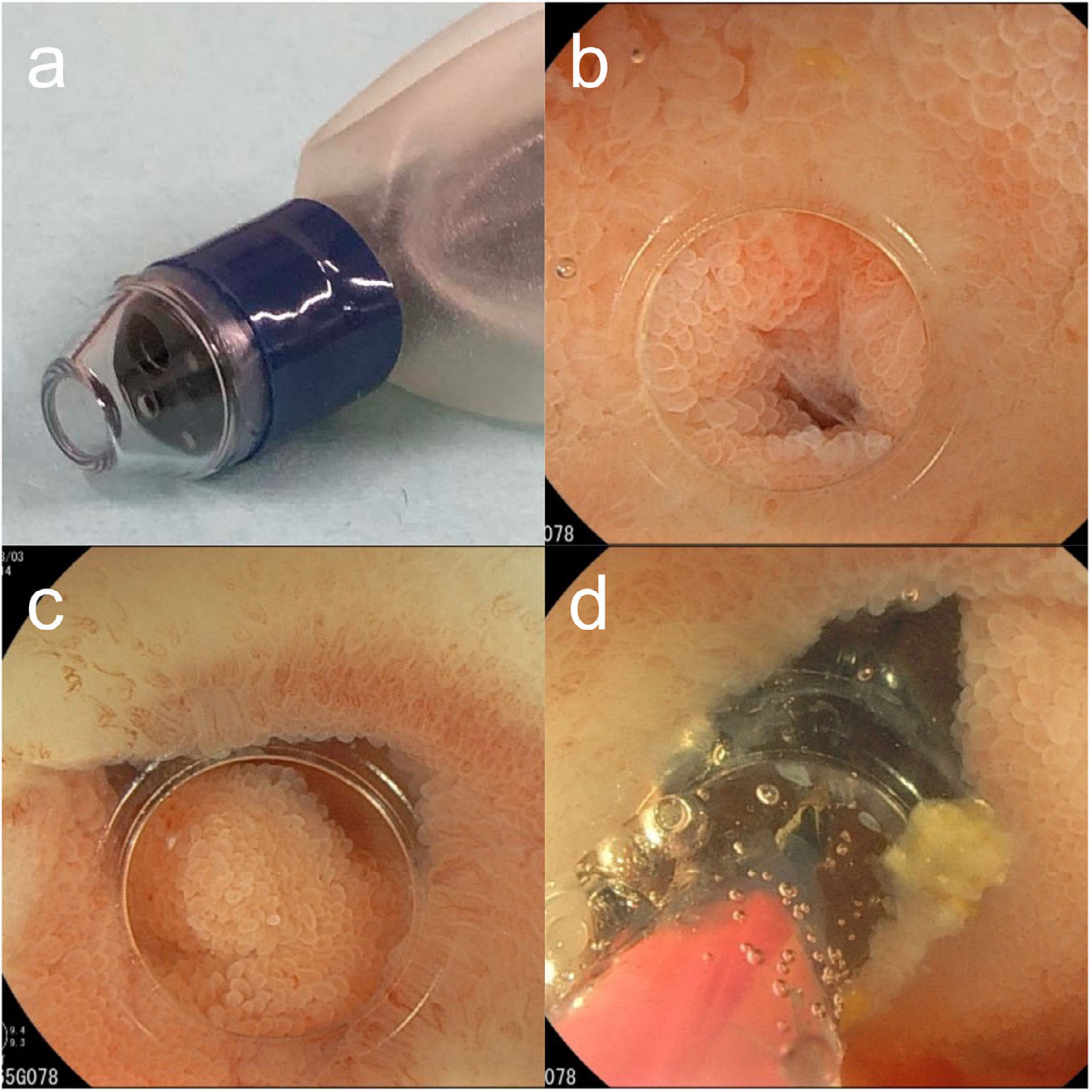
Endoscopic balloon dilation (EBD) for a small bowel stricture in Crohn's disease (CD). (a) Double‐balloon endoscope equipped with a CAST hood. (b) Small bowel stricture identified during enteroscopy. (c) Measurement of the stricture diameter using the CAST hood. A circumferential white ring is formed, allowing accurate assessment of the luminal diameter (8 mm). (d) Balloon dilation performed using a CRE dilation balloon.

Upon reaching the stricture, the morphology of the stricture is first observed to evaluate the indications for balloon dilation (Figure 5b–d). If the stricture is considered amenable to EBD, the CAST hood is wedged into the lesion to accurately assess the luminal diameter and guide balloon selection. In general, an 8–10 mm catheter is used for strictures of 4 mm or less, a 10–12 mm catheter for strictures of 4–7 mm, and a 12–15 mm catheter for strictures greater than 7 mm [57]. In most cases, a guidewire is advanced across the stricture under fluoroscopic guidance, and the dilation balloon is subsequently introduced over the wire. If guidewire advancement into the distal bowel is difficult, inflation of the distal endoscope balloon and injection of a small amount of contrast medium through a BioShield Irrigator (STERIS, OH, USA) can facilitate visualization of the bowel anatomy beyond the stricture. Instillation of 0.5 mL of olive oil into the working channel before balloon insertion can further reduce friction and improve device maneuverability. The balloon is gradually inflated to the target pressure under fluoroscopic guidance and maintained for 1 min. Dilation to 10–12 mm is generally sufficient for endoscope passage, whereas a diameter of 13.5 mm usually permits overtube passage. Nevertheless, multiple strictures remain a technical challenge for EBD and are associated with an increased risk of subsequent surgery, potentially resulting in short bowel syndrome [58]. Some reports indicate that utilizing the CAST hood allows access to deep strictures and enables the successful completion of EBD even in cases with multiple strictures [59]. After treatment of all identified strictures, endoscopic selective enteroclysis is performed to evaluate more distal lesions. During withdrawal of the endoscope, strictures that have not been dilated to the maximal intended diameter may undergo additional dilation.

### 5.4 Efficacy and Safety of Endoscopic Balloon Dilation

EBD has demonstrated an excellent success rate ranging from 74% to 100%, with clinical improvement in obstructive symptoms observed in 58%–87% of cases [47, 60]. The major clinical significance of this therapy lies in the avoidance or postponement of surgical intervention by appropriately repeating EBD [47, 49]. A 3‐year follow‐up study reported that approximately 80% of patients remained surgery‐free [61]. Conversely, symptom recurrence is observed in approximately 50% of cases, necessitating redilation [4, 47].

Regarding safety, the incidence of major complications, such as perforation and major hemorrhage, is reported to be approximately 3%–5% on a per‐patient basis and approximately 1.8%–2.6% on a per‐procedure basis [6, 49]. Perforation, the most severe complication, occurs at a rate of approximately 0%–3%. Once it occurs, emergency surgery is frequently required [49, 56]. Additionally, hemorrhage occurs at a frequency of approximately 0%–3.2%, but most cases resolve with conservative management [49]. Furthermore, as a complication specific to BAE via the transoral approach, acute pancreatitis resulting from physical stress on the duodenum and pancreas has been reported in approximately 0.3% of cases. Caution is warranted, especially when the procedure is prolonged, such as exceeding 2 h [49].

## 6 Conclusion

BAE has become an indispensable therapeutic modality for a wide range of small bowel diseases, enabling effective management of bleeding, polyposis syndromes, and Crohn's disease–associated strictures while reducing the need for surgical intervention. Recent advances in endoscopic techniques and dedicated accessories have further improved the safety and efficacy of therapeutic enteroscopy. Future developments in image‐enhanced endoscopy, device technology, and artificial intelligence–assisted lesion evaluation are expected to expand the therapeutic capabilities of BAE and further improve patient outcomes and quality of life.

## Author Contributions

Manabu Nagayama and Tomonori Yano conceived and designed the study. Manabu Nagayama drafted the manuscript. All authors critically reviewed the manuscript, contributed to its revision, and approved the final version.

## Funding

The authors have nothing to report.

## Ethics Statement

The included endoscopic images are fully anonymized without identifiable patient information; therefore, ethical approval and informed consent were not required.

## Conflicts of Interest

Tomonori Yano invented and holds patents on the dedicated gel‐immersion method and gel and receives speaker honoraria from Fujifilm, Olympus, and Otsuka Pharmaceutical Factory. Hirotsugu Sakamoto received research funding and scholarship donations from FUJIFILM Corporation/Medical Co., Ltd., and provides contractual capsule endoscopy reading support to Covidien Japan Inc.
